# Dobutamine-Induced Strain and Strain Rate Predict Viability Following Fibrinolytic Therapy in Patients with ST-Elevation Myocardial Infarction

**DOI:** 10.3389/fcvm.2015.00012

**Published:** 2015-03-11

**Authors:** Mohamed Ismail, Wail Nammas

**Affiliations:** ^1^Cardiology Department, Faculty of Medicine, Ain Shams University, Cairo, Egypt

**Keywords:** strain rate imaging, myocardial deformation indices, dobutamine stress echocardiography, myocardial infarction, viability

## Abstract

**Background:** Low-dose dobutamine stress echocardiography is increasingly used for identifying myocardial viability.

**Aim:** We explored whether dobutamine-induced strain (S) and strain rate (SR) can identify myocardial viability following fibrinolytic therapy for ST-segment-elevation myocardial infarction (STEMI), taking ^99m^Tc-sestamibi scintigraphy as the “gold standard” for diagnosis.

**Methods:** We enrolled 60 consecutive patients presenting for myocardial viability assessment at least 4 weeks following STEMI. S and SR were measured by tissue Doppler imaging individually for all myocardial segments under low-dose dobutamine stress echocardiography. Patients underwent resting ^99m^Tc-sestamibi scintigraphy using the standard imaging technique. Based on the results of ^99m^Tc-sestamibi scintigraphy, the dobutamine-induced S and SR were compared between viable and non-viable segments. Receiver-operating characteristics curve was constructed to determine the cutoff value of the dobutamine-induced S and SR that best identifies viability.

**Results:** The dobutamine-induced S and SR were significantly higher in viable compared with non-viable segments, a finding that was consistent for most individual myocardial segments (10 out of 16 for S and 11 out of 16 for SR). A cutoff value ranging from −8.5 to −9.6% for the S identified viability in apical and mid- segments, whereas a cutoff value ranging from −11.5 to −21.5% identified viability in basal segments. Similarly, a cutoff value ranging from −0.5 to −1.2 s^−1^ for the SR identified viability in apical and mid-segments, whereas a cutoff value ranging from −1.4 to −1.7/s^−1^ identified viability in basal segments.

**Conclusion:** In patients undergoing viability assessment following fibrinolytic therapy for STEMI, the dobutamine-induced S and SR were higher in viable versus non-viable segments. A cutoff value of dobutamine-induced S and SR identified viability in most individual myocardial segments.

## Introduction

Identification of viable myocardium following acute myocardial infarction has gained crucial importance with the recent step-up in myocardial revascularization techniques. The amount of viable myocardium is a “surrogate” of future improvement of left ventricular systolic function: the most powerful single predictor of long-term prognosis (1). Traditionally, evaluation of regional myocardial function was performed by visual assessment of thickening and inward displacement of individual myocardial segments. Limited by operator dependency and poor visualization of some myocardial segments, it was largely supplanted by estimation of tissue velocities, using tissue Doppler imaging (TDI). However, this technique is influenced by whole heart translation and tethering movement from adjacent myocardial segments (2). Eventually, assessment of myocardial deformation indices circumvented these limitations. Evidence supports the superiority of strain (S) and strain rate (SR) measurement over tissue velocity by TDI for the evaluation of regional myocardial function (3–5).

Low-dose dobutamine stress echocardiography (DSE) is well-acknowledged for identifying viable myocardium. Dobutamine-induced wall motion improvement is specific for predicting reversible contractile dysfunction; however, its sensitivity is suboptimal (6). Yet, the value of dobutamine-induced myocardial deformation indices (S and SR) for detection of myocardial viability remains unclear. Therefore, we explored whether dobutamine-induced S and SR can identify myocardial viability following fibrinolytic therapy for ST-segment-elevation myocardial infarction, taking ^99m^Tc-sestamibi scintigraphy as the “gold standard” for diagnosis.

## Materials and Methods

### Patient selection and study design

Prospectively, we enrolled 60 consecutive patients who presented to our nuclear cardiology unit for myocardial viability assessment, at least 4 weeks following ST-segment-elevation myocardial infarction, during the period from May 2012 to May 2013. All patients had received fibrinolytic therapy (streptokinase 1,500,000 U, given by intravenous infusion over 30–60 min), that started within 12 h of the onset of chest pain. Patients were considered eligible for inclusion if they had regional wall motion abnormality in the anatomical distribution of the infarct zone as detected by resting 2-D echocardiography. We excluded patients with early post-infarction unstable angina or severe hemodynamic instability, clinically evident congestive heart failure, significant valvular or congenital heart disease, any myocardial disease apart from ischemia, atrial fibrillation, bundle branch block, and technically inadequate echocardiographic imaging defined as more than two non-analyzable segments in the infarct zone. Similarly, we excluded from analysis segments with poor stain or strain rate signal (due to low signal-to-noise ratio or reaching aliasing velocity). Before inclusion, informed written consent was obtained from each patient after full explanation of the study protocol. Finally, the study protocol was reviewed and approved by the institutional Human Research Committee, as it conforms to the ethical guidelines of the 1964 Declaration of Helsinki, as revised in 2013.

### Baseline echocardiographic assessment

Assessment of regional and global left ventricular systolic function was performed by trans-thoracic echocardiography, using a General Electric Vivid 7 Pro cardiac ultrasound machine (GE Medical Systems, Horten, Norway) equipped with harmonic imaging capabilities. A 3.5 MHz phased-array transducer was used to obtain standard 2-D, M-mode, Doppler flow, and TDI. Patients were examined in the left lateral recumbent position using standard parasternal and apical views. Global left ventricular systolic function was assessed in apical 2- and 4-chamber views using the biplane Simpson’s method. Regional wall motion was assessed according to the standard 16-segment model recommended by the American Society of Echocardiography (7).

### Strain and strain rate imaging

Strain color imaging was performed one wall at a time in order to achieve a frame rate above 140 frames/s, and so that the angle between the Doppler beam and the longitudinal shortening direction of the wall was kept below 30°. Systolic S and SR measurement were performed for all analyzable segments according to the standard 16-segment model. An offset to measure S and SR was set at 12 mm. The stationary region of interest was 10 mm in longitude, and 6 mm in latitude, centered in the middle of the targeted myocardial segment. Systolic time to measure systolic S and SR was determined from the electrocardiographic tracing as the time from the peak of the R wave to the end of the T wave. Peak systolic S was determined as the maximal negative S at the end of systole. Peak systolic SR was determined as the maximal negative SR within 350 ms after the onset of systole. Measurements were obtained from three cardiac cycles and an average was taken. Cardiac cycles associated with extra-systolic, post-extra-systolic beats, or any other rhythm disturbances were excluded from analysis.

### Stress echocardiographic protocol

**Figure 1:**
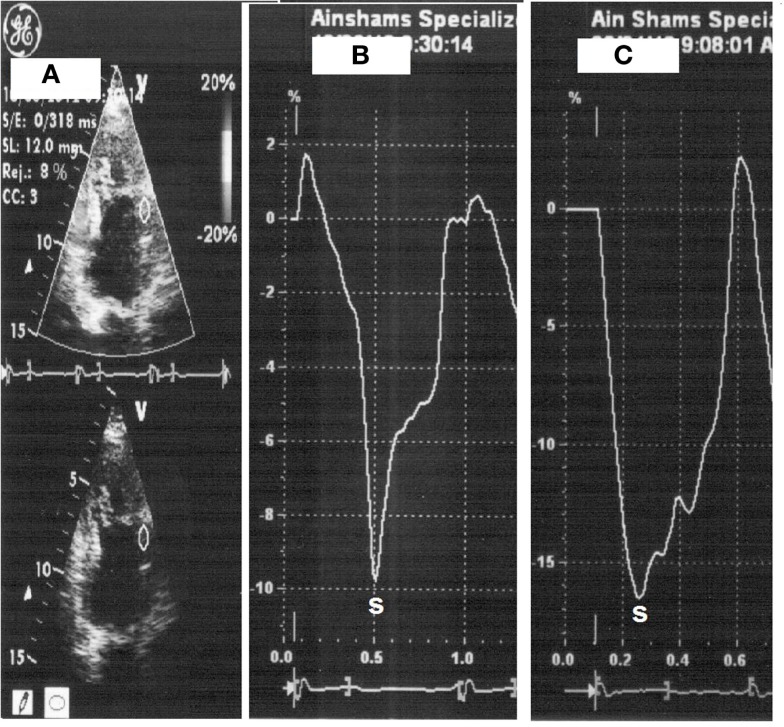
**Strain measurement with placement of sample on the mid-anterior wall segment (A), strain at rest (heart rate 66 beats/min) (B) then with dobutamine (heart rate 110 beats/min) (C)**.

**Figure 2:**
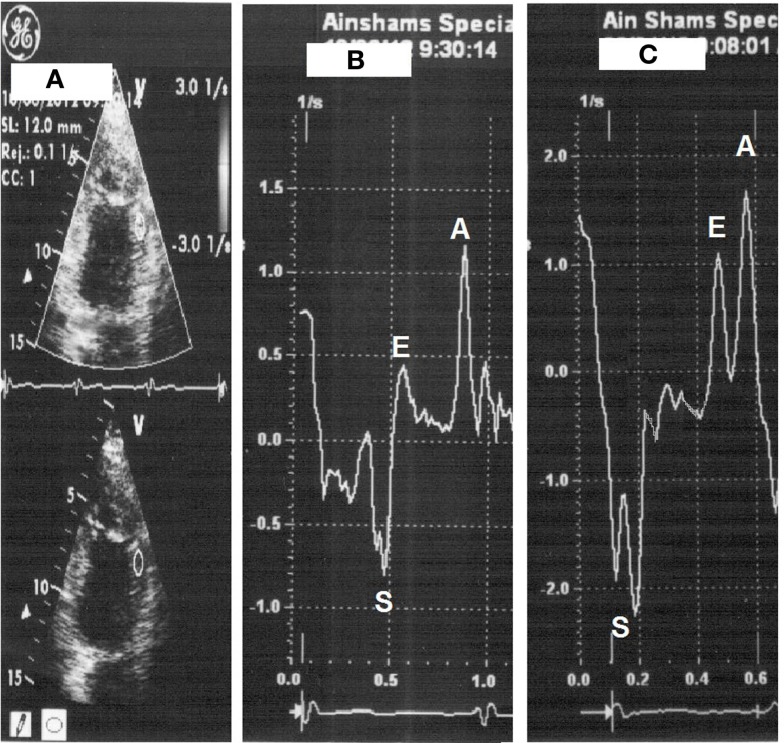
**Strain rate measurement with placement of sample on the mid-anterior wall segment (A), strain rate at rest (heart rate 66 beats/min) (B) then with dobutamine (heart rate 112 beats/min) (C)**.

All patients underwent low-dose DSE as follows: dobutamine (Dobutrex^®^, Lilly, Eli and Company, Indianapolis, IN. USA) was infused intravenously starting at 5 μg/kg/min, increased up to 20 μg/kg/min in 3-min stages. Standard views were recorded at baseline, as well as at the end of the infusion protocol. Images were digitized in cine-loop format and saved for subsequent playback and analysis. Views were analyzed offline by a single observer (Mohamed Ismail) employing the software program of the echocardiography machine (Echopac PC, GE Vingmed Ultrasound, GE Medical Systems). Systolic S and SR measurements were performed individually for each segment, both at baseline and at the end of dobutamine infusion (Figures 1 and 2). The stress test was performed with the patients on their full anti-ischemic medications.

### ^99m^Tc-sestamibi scintigraphy imaging protocol

Patients underwent resting ^99m^Tc-sestamibi imaging study with the administration of trimetazidine, using the standard imaging technique. Trimetazidine (Vastarel^®^, Servier, France) was administered by the oral route the day before the study (60 mg in three divided equal doses 8 h apart), and 1 h before performing the study (60 mg single dose). Sublingual nitroglycerin 0.8 mg was administered in two divided doses with a 5-min interval just before radioactive tracer administration. The second dose was not given if the heart rate increased by 10 beats/min or more, or the systolic blood pressure decreased by 10 mm Hg or more after the first dose. Injection of 20–25 mCi of radioactive tracer was performed 45–60 min before SPECT image acquisition. Images were acquired using a rotating single-head gamma camera (GE Medical Systems, Starcam 4000i, UK) equipped with low-energy all-purpose collimators. Energy windows of 20% were respectively centered on the 140-keV peaks of ^99m^Tc-sestamibi. Thirty-two images were obtained over 180° extending from the 45° right anterior oblique to the 45° left posterior oblique projections, using a 64 × 64 acquisition matrix. All studies were subjected to quality-control checks and corrections when necessary for camera non-uniformity, center-of-rotation offsets, patient motion, and “upward creep.”

### ^99m^Tc-sestamibi scintigraphy image analysis

Two experienced nuclear cardiologists blinded for both clinical and TDI data analyzed the scintigraphy images. Trans-axial reconstruction was performed using the standard back projection technique with a Ramp-Hanning filter. The reconstructed tomographic slices were 6 mm thick and reoriented along the short, horizontal long-, and vertical long-axis for interpretation. The vascular assignment of myocardial segments to the conventional anatomic distribution of major coronary arteries was performed according to the 17-segment Scoring System (8). Images were interpreted by visual semi-quantitative analysis. Segmental ^99m^Tc-sestamibi uptake was scored using the 5-point Scoring System as follows:

- 0 = normal uptake,
- 1 = mildly reduced uptake,
- 2 = moderately reduced uptake,
- 3 = severely reduced uptake,
- 4 = absent uptake.

Patterns of viability were based on the segmental radioactive tracer uptake so that segments were then individually classified into viable or non-viable. Score of 0 was considered as normal; 1 and 2 as viable; 3 and 4 as non-viable.

### Statistical analysis

Continuous variables were presented as mean ± SD, if they were normally distributed. Data were tested for normal distribution using the Kolmogorov–Smirnov test. Categorical variables were described with absolute and relative (%) frequencies. According to the above assignment of myocardial segments, dobutamine-induced S and SR values were compared between viable and non-viable groups in each individual anatomical segment by means of the unpaired *t*-test. Eventually, we generated receiver-operating characteristics curve to identify the cutoff value of the dobutamine-induced S and SR that best discriminates viable from non-viable myocardial segments based on ^99m^Tc-sestamibi scintigraphy. The optimal cutoff value was defined as the value giving the largest area under the curve. Finally, ten cases were randomly selected for analysis of intra-observer variability of repeated measurement by the single observer. Assessment of variability was performed using linear regression. All analyses were two-sided and a probability value of *p* < 0.05 was considered statistically significant. Analyses were performed with SPSS version 12.0 statistical package (SPSS Inc., Chicago, IL, USA).

## Results

A total of 60 consecutive patients presenting at least 4 weeks following ST-segment-elevation myocardial infarction were enrolled in the current study. Baseline characteristics of the study cohort are presented in Table 1. The mean age was 54.8 ± 10.9 years; 85% were males. Analysis of TDI-measured S and SR was feasible in 954 segments (99.4%) of all 960 segments. These included 397 (41.4%) segments defined as dyssynergic, and 557 (58%) defined as normal by resting 2-D echocardiography. Among 397 (41.4%) defined as dyssynergic at rest, 209 (21.8%) were assigned as severely hypokinetic, 188 (19.6%) as akinetic or dyskinetic. Six segments (0.6%) were considered as not feasible for S and SR measurement, and thus were excluded from analysis. This was due to either low signal-to-noise ratio, or aliasing in the velocity dataset that was not perceived during image acquisition. ^99m^Tc-sestamibi scintigraphy image analysis was feasible in all segments.

**Table 1:** **Baseline clinical characteristics of the cohort**.

	Total cohort (*N* = 60)
Age (years)	54.8 ± 10.9
Male: gender	51 (85)
Diabetes mellitus	26 (43.3)
Hypertension	29 (48.3)
Smoking	43 (71.7)
Dislipidemia	13 (21.7)
Family history of IHD	18 (30)
2-D Ejection fraction (%)	35 ± 8
Left ventricular EDD (mm)	62 ± 7
Left ventricular ESD (mm)	49 ± 5
Left atrial dimension (mm)	46 ± 7

*Continuous variables are presented as mean ± SD, whereas categorical variables are presented as numbers (%)*.

*IHD, ischemic heart disease; EDD, end-diastolic dimension; ESD, end-systolic dimension*.

Dobutamine-induced S was significantly higher in viable, compared with non-viable segments in the following individual myocardial segments: mid- and apical lateral, basal and apical posteroseptal, mid- and apical anterior, basal and mid-anteroseptal, basal and mid-posterior (*p * < 0.05 for all) (Table 2). Similarly, dobutamine-induced SR was significantly higher in viable, compared with non-viable segments in the following individual myocardial segments: mid- and apical lateral, basal and apical posteroseptal, mid- and apical anterior, apical inferior, basal and mid-anteroseptal, basal and mid-posterior (*p * < 0.05 for all) (Table 3).

**Table 2:** **Dobutamine-induced strain values in individual myocardial segments**.

Segment	Viable	Non-viable	*p* Value
Basal lateral	−15.9 ± 5.2	NA	NA
Mid-lateral	−13.9 ± 4.9	−7.4 ± 3.2	0.00
Apical lateral	−11.4 ± 4.8	−7.7 ± 3.7	0.00
Basal posteroseptal	−25.8 ± 10.4	−13.6 ± 6.8	0.00
Mid-posteroseptal	−13.3 ± 4.5	−12.7 ± 4.2	0.86
Apical posteroseptal	−14.4 ± 4.5	−4.7 ± 7.2	0.00
Basal anterior	−21.1 ± 8.0	NA	NA
Mid-anterior	−14.0 ± 6.2	−5.7 ± 2.5	0.00
Apical anterior	−13.5 ± 8.4	−5.6 ± 2.1	0.00
Basal inferior	−19.4 ± 7.8	−17.9 ± 5.9	0.63
Mid-inferior	−9.2 ± 7.1	−7.1 ± 6.2	0.12
Apical inferior	−8.9 ± 6.7	−5.6 ± 4.7	0.26
Basal anteroseptal	−21 ± 12.8	−7.8 ± 4.8	0.00
Mid-anteroseptal	−12.0 ± 5.4	−8.5 ± 6.1	0.04
Basal posterior	−14.6 ± 6.8	−8.0 ± 3.3	0.00
Mid-posterior	−11.7 ± 7.2	−7.4 ± 3.6	0.01

*All values are expressed as %*.

**Table 3:** **Dobutamine-induced strain rate values in individual myocardial segments**.

Segment	Viable	Non-viable	*p* Value
Basal lateral	−1.7 ± 0.9	NA	NA
Mid-lateral	−1.3 ± 0.1	−0.37 ± 0.7	0.00
Apical lateral	−1.3 ± 0.6	−0.5 ± 0.3	0.00
Basal posteroseptal	−2.1 ± 1.4	−1.1 ± 0.8	0.04
Mid-posteroseptal	−1.8 ± 0.6	−1.4 ± 0.9	0.09
Apical posteroseptal	−1.4 ± 0.6	−0.6 ± 0.5	0.00
Basal anterior	−2.0 ± 0.9	NA	NA
Mid-anterior	−1.3 ± 0.4	−0.4 ± 0.5	0.00
Apical anterior	−1.8 ± 1.2	−0.5 ± 0.3	0.00
Basal inferior	−1.3 ± 0.4	−1.2 ± 0.5	0.14
Mid-inferior	−0.8 ± 0.7	−0.9 ± 0.5	0.23
Apical inferior	−1.4 ± 0.1	−0.4 ± 0.3	0.00
Basal anteroseptal	−2.0 ± 0.8	−0.9 ± 0.2	0.00
Mid-anteroseptal	−1.3 ± 0.6	−0.9 ± 0.2	0.04
Basal posterior	−1.5 ± 0.9	−0.9 ± 0.4	0.04
Mid-posterior	−1.8 ± 0.7	−0.8 ± 0.4	0.00

*All values are expressed as s^−1^*.

Receiver-operating characteristics curve analysis identified the optimal cutoff value of the dobutamine-induced S and SR that best discriminates viability – from non-viability – in the above mentioned segments. Taking ^99m^Tc-sestamibi scintigraphy as the “gold standard” for diagnosis, a cutoff value ranging from –8.5 to –9.6% for the S identified viability in apical and mid- segments, whereas a cutoff value ranging from –11.5 to –21.5% identified viability in basal segments (Table 4). Similarly, a cutoff value ranging from –0.5 to –1.2/s^−1^ for the SR identified viability in apical and mid- segments, whereas a cutoff value ranging from –1.4 to –1.7/s^−1^ identified viability in basal segments (Table 5).

**Table 4:** **Cutoff values for dobutamine-induced strain that predict viability in individual myocardial segments**.

Segment	Cutoff value	Sensitivity (%)	Specificity (%)
Mid-lateral	−8.7	83.3	86.2
Apical lateral	−8.5	63.6	85.7
Basal posteroseptal	−21.5	100	61.3
Apical posteroseptal	−8.8	81.2	89.3
Mid-anterior	−9.7	100	82.7
Apical anterior	−8.5	86.7	66.7
Basal anteroseptal	−11.5	88.9	94.9
Mid anteroseptal	−9.8	87.3	77.6
Basal posterior	−10.9	84.2	79.6
Mid-posterior	−9.2	68	72.4

*All values are expressed as %*.

**Table 5:** **Cutoff values for dobutamine-induced strain rate that predict viability in individual myocardial segments**.

Segment	Cutoff value (%)	Sensitivity (%)	Specificity (%)
Mid-lateral	−0.5	83.3	100
Apical lateral	−0.8	79.5	78.6
Basal posteroseptal	−1.4	77.8	67.7
Apical posteroseptal	−0.8	68.8	85.7
Mid-anterior	−0.6	75	90.4
Apical anterior	−0.9	91.1	80
Apical inferior	−1.1	93.9	72.7
Basal anteroseptal	−1.5	100	74.4
Mid-anteroseptal	−1.1	94.1	43.9
Basal posterior	−1.4	100	43.8
Mid-posterior	−1.1	84	89.7

*All values are expressed as s^−1^*.

The DSE protocol was well-tolerated by all patients with no major side effects during or after the test. The intra-class correlation between repeated measurements by the single observer (M.I.) was *r* = 0.91 (95% confidence interval 0.86–0.95) for the S, and *r* = 0.93 (95% confidence interval 0.88–0.98) for the SR.

## Discussion

### Main findings

The current study demonstrated that in patients undergoing viability assessment following fibrinolytic therapy for ST-segment-elevation myocardial infarction, the dobutamine-induced S and SR were significantly higher in viable segments, compared with non-viable ones, a finding that was consistent for most individual myocardial segments (10 out of 16 for S and 11 out of 16 for SR).

### Deformation indices for detection of viability

DSE is known to be operator-dependent; and therefore, liable for high inter- and intra-observer variability. Nevertheless, being feasible, safe, and inexpensive, with a fairly high diagnostic and prognostic accuracy, low-dose DSE is widely acknowledged for evaluation of viability following myocardial infarction. In the quest to overcome the subjective visual assessment of regional contractility, a good body of research has rigorously pursued to adopt quantitative measures of regional myocardial function during low-dose DSE. Although appealing at the outset, TDI-derived systolic tissue velocity was reportedly overwhelmed by “tethering” effect from neighboring segments, as well as by whole heart translation movement (2). Recently, some studies have explored the potential of myocardial deformation indices for the evaluation of myocardial viability (9–12). A recent report demonstrated an incremental value of TDI-based S and SR during low-dose DSE over wall motion analysis in the prediction of functional recovery following revascularization (13). In another study, longitudinal S and SR derived from speckle-tracking echocardiography under DSE demonstrated a similar sensitivity and a higher specificity for detection of viability following myocardial infarction, compared with SPECT (14). However, neither of these latter studies addressed the issue of identifying a cutoff value for the dobutamine-induced S and SR that best predicts viability in individual myocardial segments.

Going a step further, we explored the theme of “which level” of dobutamine-induced S and SR would identify viability in individual myocardial segments. In this sense – and to the best of the authors’ knowledge – the current study was the first to identify cutoff values of the dobutamine-induced S and SR that best recognize the viable segments on individual segment basis. Moreover, our results serve to support the results obtained by the prior studies, adding more strength to the “level of evidence.” Unfortunately, however, the results were somewhat inconsistent; favorable for many segments, but disappointing for some. The lack of consistency of the results in both S and SR might be attributed to angle dependency, a well-known downside of the strain imaging technique. Failure to keep the angle between the Doppler beam and the longitudinal shortening direction of the segment below 30° may have precluded accurate measurement of S and SR in some segments.

We adopted measurement of TDI-based S and SR for assessment of myocardial viability by low-dose DSE. TDI-based measures have been constrained by susceptibility to signal noise and dependence on the angle of insonation (2). The recently introduced speckle-tracking echocardiography has no angle dependency, but it needs adequate image quality and operates at a limited frame rate (15, 16). Nevertheless, in a recent report by Bansal et al., comparing the two modalities for predicting myocardial viability under low-dose DSE, TDI-based measures were more accurate, and predicted viability in both the anterior and posterior circulation; whereas speckle-tracking echocardiography measures predicted viability only in the anterior circulation (13).

### ^99m^Tc-sestamibi scintigraphy for detection of viability

Since we adopted ^99m^Tc-sestamibi scintigraphy as the “gold standard” for diagnosis of viability, we sought to improve its diagnostic accuracy. Thus, we employed a test protocol that entails the administration of both trimetazidine and nitroglycerin before radiotracer injection. A recent study by Feola et al. showed that the addition of trimetazidine to ^99m^Tc-tetrofosmin scintigraphy improved the sensitivity of the perfusion scan for the detection of viable myocardial tissue, both at 2- and 6-months follow-up, compared with placebo, maintaining a satisfactory specificity (17). Furthermore, the predictive power of ^99m^Tc-sestamibi scintigraphy was also enhanced with the addition of nitrates, obtaining an improvement in both the sensitivity and specificity (up to 95 and 88%, respectively) (18).

### Clinical implications

In many centers, low-dose DSE remains the mainstay for detection of myocardial viability. Identification of viability following myocardial infarction is of paramount importance for the decision to triage patients for revascularization procedures. Nevertheless, the problems of needed expertise and lack of reproducibility have always put low-dose DSE under intense scrutiny. In a trial to improve the credibility of the widely popular test for viability detection, an “objective” way of assessment is eagerly needed. In this context, measurement of myocardial deformation indices during low-dose DSE would offer a substantial upgrading for the test. Moreover, the identification of cutoff values for the dobutamine-induced S and SR for the individual myocardial segments would further present a palatable easy-to-perform way to discriminate myocardial segments whose function will “recover” following revascularization. Going this way, low-dose DSE would potentially become the “standard of care” for viability detection in a not-too-distant future.

### Limitations of the study

Our findings were based on a single center study with a relatively small sample size of the cohort, a fact that makes it difficult to generalize our results to all patients undergoing viability assessment. Multicenter studies using the same protocol and examining a larger number of patients are needed. Additionally, the current technology of SR imaging is characterized by considerable noise in the SR signal. This noise increases further with higher heart rates and reduces image quality. Another limitation of the technique is the angle-dependency, which makes determination of S and SR in some segments less accurate. The adoption of a frame rate above 140 frames/s is another limitation. Furthermore, ECG tracking was used to detect systolic time rather than aortic valve opening and closure which is more accurate. Another limitation is that all patients received fibrinolytic therapy, and none of the patients was treated by primary percutaneous coronary intervention. It would be of interest to see if primary percutaneous coronary intervention shows greater viability in myocardial segments compared with fibrinolysis. Finally, we adopted ^99m^Tc-sestamibi scintigraphy as the “gold standard” for diagnosis of viability. Actual improvement of myocardial segment contractility following revascularization would offer a more “real” gold standard for comparison.

## Conclusion

In patients undergoing viability assessment following fibrinolytic therapy for ST-segment-elevation myocardial infarction, the dobutamine-induced S and SR were higher in viable compared with non-viable segments. A cutoff value of dobutamine-induced S and SR identified viability in most individual myocardial segments.

## Conflict of Interest Statement

The authors declare that the research was conducted in the absence of any commercial or financial relationships that could be construed as a potential conflict of interest.
